# A Non-Cytotoxic Resin for Micro-Stereolithography for Cell Cultures of HUVECs

**DOI:** 10.3390/mi11030246

**Published:** 2020-02-26

**Authors:** Max J. Männel, Carolin Fischer, Julian Thiele

**Affiliations:** Leibniz-Institut für Polymerforschung Dresden e.V., Hohe Str. 6, 01069 Dresden, Germany; maennel@ipfdd.de (M.J.M.); fischer-carolin@ipfdd.de (C.F.)

**Keywords:** micro-stereolithography, cytotoxicity, resin development, microfluidics, cell culture, human umbilical vein endothelial cells (HUVECs), biocompatibility, 3D printing, additive manufacturing

## Abstract

Three-dimensional (3D) printing of microfluidic devices continuously replaces conventional fabrication methods. A versatile tool for achieving microscopic feature sizes and short process times is micro-stereolithography (µSL). However, common resins for µSL lack biocompatibility and are cytotoxic. This work focuses on developing new photo-curable resins as a basis for µSL fabrication of polymer materials and surfaces for cell culture. Different acrylate- and methacrylate-based compositions are screened for material characteristics including wettability, surface roughness, and swelling behavior. For further understanding, the impact of photo-absorber and photo-initiator on the cytotoxicity of 3D-printed substrates is studied. Cell culture experiments with human umbilical vein endothelial cells (HUVECs) in standard polystyrene vessels are compared to 3D-printed parts made from our library of homemade resins. Among these, after optimizing material composition and post-processing, we identify selected mixtures of poly(ethylene glycol) diacrylate (PEGDA) and poly(ethylene glycol) methyl ethyl methacrylate (PEGMEMA) as most suitable to allow for fabricating cell culture platforms that retain both the viability and proliferation of HUVECs. Next, our PEGDA/PEGMEMA resins will be further optimized regarding minimal feature size and cell adhesion to fabricate microscopic (microfluidic) cell culture platforms, e.g., for studying vascularization of HUVECs in vitro.

## 1. Introduction

Microfluidics is a versatile tool to confine fluids in microscopic channel networks and to control flow pattern formation therein. Over the last two decades, the fabrication of microfluidic devices has been dominated by glass microcapillary assembly as well as a combination of photo- and soft lithography utilizing poly(dimethylsiloxane) (PDMS) [1,2]. While PDMS-based microfluidics has been largely used to fabricate microchannel networks with uniform height, the desired flow pattern formation of multiphase flow inside these devices mostly requires post-processing and localized functionalization, e.g., to tailor microchannel surface wettability. Inspired by coaxial flow formation of multi-phase flow in glass microcapillaries that largely reduces the need for wettability patterning, several approaches have been published to form a similar flow pattern in PDMS-based microfluidics [3,4,5]. However, these usually involve multi-layered flow cell fabrication, and thus precise alignment of all layers, which demands significant experience by experimentalists.

To overcome the limitations of conventional planar microchannel architecture, 3D printing has moved into the focus of research. Several techniques have been applied to print microfluidic devices such as fused deposition modeling [6], inkjet printing [7], two-photon polymerization [8], and micro-stereolithography (µSL) [9]. In particular, µSL is of enormous interest due to the short fabrication time (<1 h), high-resolution (<100 µm), and the reliability of the fabrication process. In µSL, low molecular weight and photo-curable resins are polymerized by ultraviolet (UV)-light in a layer-by-layer process yielding complex 3D microstructures. A rapidly increasing number of publications demonstrates the accessibility and interest in the fabrication of polymer microstructures, and microfluidic devices in particular, utilizing µSL [10,11,12,13]. As a material basis, both homemade printers and resins have been employed. For instance, the Nordin group showed the fabrication of microfluidic components such as valves and pumps via µSL [14], and recently, Thiele and coworkers demonstrated the fabrication of non-planar droplet makers for (double) emulsion formation [9]. While Nordin and coworkers used a homemade printer and resin for their studies, we utilized both a commercial resin and a commercially available printer. However, in-depth knowlegth on the composition of commercial resins is usually not available, therefore it is challenging to alter given material properties in a controlled fashion.

As conventional PDMS-based microfluidic devices have been designed to serve as blood or single-cell analysis platforms as well as biomedical devices early on, it is crucial to ensure bio- and cytocompatibility as well as non-genotoxicity of newly designed resins to implement a similar level of applicability and versatility in µSL-based 3D printing of microfluidics. Along these lines, several research groups have looked into the biocompatibility of resins in their studies, e.g., for seeding human mesenchymal stem cells in manufactured scaffolds using a µSL system [15]. Furthermore, Folch et al. used commercially available WaterShed resin coated with Matrigel to cultivate C2C12 myoblast cells on it [16], and in another example, poly-l-lysine was coated onto µSL-printed resin surfaces to improve cell attachment [17].

In this context, we introduce a library of homemade resins based on poly(ethylene glycol) diacrylate (PEGDA), poly(ethylene glycol) methyl ether methacrylate (PEGMEMA), tri(propylene glycol) diacrylate (TPGDA), and 2-phenoxyethyl acrylate (POEA) for fabricating biocompatible 3D-printed polymer materials. By focusing on homemade resins rather than utilizing commercially available ones, we have full control over material properties such as polymerization depth, contact angle, swelling behavior, and surface roughness. We then investigate the cytotoxic effect of 3D-printed parts prepared from seven different resins on human umbilical vein endothelial cells (HUVECs). By introducing post-processing steps that include washing with surfactants (Tween 20, Triton X-100, Sigma Aldrich, St. Louis, MO, USA) in phosphate-buffered saline (PBS) before cell culturing, we ensure that unbound or unconsumed components of the initial resin such as the base material, the ultraviolet (UV)-absorber, and the photo-initiator, do not leak out from the µSL-printed material during long-term cell culturing, potentially harming the cells. We show that HUVECs grow in the presence of 3D-printed materials made from three different resins based on PEGDA and PEGMEMA for more than 3 weeks and are similar in shape and size to cells grown in a poly(styrene) (PS) standard culturing environment. Finally, we measure the metabolic activity of HUVECs to obtain information about their viability when being cultured on the surface of the same 3D-printed materials. This work contributes to the design of cytocompatible polymer materials by µSL utilizing homemade resins, and thus to the future fabrication of microfluidic devices for biosciences by high-resolution 3D printing.

## 2. Materials and Methods

### 2.1. Materials

1,3,5-Triallyl-1,3,5-triazine-2,4,6(1*H*,3*H*,5*H*)-trione (TATATO), diphenyl(2,4,6-trimethylbenzoyl)phosphine oxide (TPO), phosphate-buffered saline (PBS), poly(ethylene glycol) diacrylate (PEGDA, $\bar{M_{w}}$ = 250 and 575 g mol^−1^), penicillin, poly(ethylene glycol) methyl ether methacrylate (PEGMEMA, $\bar{M_{w}}$ = 500 g mol^−1^), streptomycin, Sudan 1, tri(propylene glycol) diacrylate (TPGDA; a mixture of isomers, $\bar{M_{w}}$ = 300.35 g mol^−1^), Triton X-100, Tween 20 and trypsin/EDTA solution were purchased from Sigma Aldrich (St. Louis, MO, USA). 2-Phenoxyethyl acrylate (POEA) was bought from TCI Deutschland GmbH (Eschborn, Germany). DARQ5™ (5 mM) was received from Biostatus (Shepshed, UK). HUVEC culture medium was supplied by PromoCell (Heidelberg, Germany). We obtained 48-well and 6-well CELLSTAR suspension culture plates from Greiner Bio-One (Kremsmünster, Austria). Culture flasks (T-75) were purchased from TPP (Trasadingen, Switzerland). 1-Propanol (isopropyl alcohol, IPA) was supplied by Fisher Chemical (Pittsburgh, PA, USA). KIMTECH Science Precision Wipes were bought from Kimberly-Clark (Dallas, TX, USA). Hoechst 33342 dye and PrestoBlue™ Cell Viability Reagent were supplied by Invitrogen (Carlsbad, CA, USA). Phalloidin ATTO 550 and phalloidin ATTO 688 were received from ATTO Technology (Amherst, NY, USA). Glycine and paraformaldehyde were bought from Merck (Darmstadt, Germany). Fetal bovine serum was purchased from Biochrom (Berlin, Germany). Deionized water was prepared in a Milli-Q Direct 8 instrument (Merck Millipore, Darmstadt, Germany) with a resistivity of 18.2 MΩ cm^−1^.

### 2.2. Resin Formulation

The photo-curable resins consist of a monomer (oligomer), a crosslinker, a UV-absorber, and a photo-initiator. To develop a resin suitable for (cell-)biological applications, several resin compositions (RC) were studied (Table 1). TATATO was added as a crosslinker to the RCs containing monofunctional and difunctional base material (RC2, RC4, RC5, RC8, and RC9) to increase the crosslinking density [18]. As a photo-initiator and UV-absorber, TPO and Sudan 1, respectively, were chosen due to the overlap of their respective absorbance spectra with the light-emitting diode (LED) of the 3D printer (λ = 385 nm). The initiator concentration was kept to 0.1% (*w/w*) except for RC5. Here, it is set to 0.25% (*w/w*) since methacrylates are known to be less reactive than acrylates [19].

### 2.3. Three-Dimensional (3D) Printing via Micro-Stereolithography (µSL)

Autodesk Inventor Professional 2019 (Autodesk, San Rafael, CA, USA) was used to design all polymer material parts 3D-printed in this work. The designs are subsequently converted into a standard tessellation language (STL) file by the software and loaded into the ASIGA composer software. Two digital light projector (DLP)-based printers (Asiga PICO2^HD^ and PICO2; ASIGA, Alexandria, Australia) are used for resin processing and additive manufacturing, respectively. Different printing parameters were adjusted in the composer software and provided control over layer thickness, separation distance, and exposure time. After the printing process, all objects were washed with IPA to remove excess resin followed by post-curing the object for 300 s with 10 UV-light flashes per second using an Otoflash G171 chamber (NK-Optik GmbH, Baierbrunn, Germany). Following this procedure, the printed parts were ready for use.

### 2.4. Dose Calibration

To evaluate the exposure time required for different layer thicknesses, spots with an approximated diameter of 3 mm were printed by exposing uncured resin with UV-light with an intensity of 16.3 mW cm^−2^. Using a confocal microscope (Nanofocus Expert and µsoft metrology software for analysis, Nanofocus AG Oberhausen, Germany), the thicknesses of the spots were determined, and the corresponding exposure energy *E*_exp_ was calculated using the following equation:

(1) $$E_{\exp}=Ixt_{\exp}$$

Here, *I* is the intensity of the UV-lamp and *t*_exp_ the exposure time. By plotting the layer thickness of the spot against the exposure energy, a linear fit can be applied, and the energy for a certain layer thickness can be calculated.

### 2.5. Contact Angle Measurements

The wettability of water toward polymer materials printed with the resins RC1 to RC9 and PS as a standard reference was determined using an OCA instrument (Dataphysics Instruments GmbH, Filderstadt, Germany). Blocks (30 mm × 20 mm × 5 mm in length, depth, and height, respectively) were printed using a layer thickness of 50 µm for each resin. By wiping the parts with dust-free wipes and drying them with pressurized air, a dust-free and water-free surface was provided, which is mandatory for contact angle measurements. To study the wettability, the advancing contact angle was measured for a sessile droplet with the needle-in-drop method. Using the OCA Software SCA20 (Version 2), the recorded droplets were analyzed. To ensure sufficient statistics, 10 droplets with a volume of 5 µL each and a volume dosing rate of 0.25 µL s^−1^ was measured per sample. The average contact angle was then calculated by applying a tangential fit to the individual droplets.

### 2.6. Surface Roughness

It is known that the surface roughness of a substrate influences cell culture conditions [20], e.g., vascular tissue showed higher response on rough surfaces (*R_a_* > 1 µm) compared to smooth surfaces (*R_a_* < 1 µm). To evaluate the surface roughness of our 3D-printed objects, a confocal microscope (Nanofocus) was used according to ISO 4287. For each sample, 10 cross-sections over 1500 µm were measured using a 10-fold magnification, where five cross-sections were vertical and five were horizontal, as sketched in Figure S1.

### 2.7. Swelling of Printed Parts in Buffer

The swelling of 3D-printed objects was measured using the scanner FLA-5100 (FUJIFILM, Minato, Japan) and Multi Gauge software (Version 3.1, FUJIFILM, Minato, Japan). The swelling ratio was then calculated as:

(2) $$swelling\;ratio\;\left[\%\right]=\frac{D_{\mathrm{swollen}}-D_{\mathrm{dry}}}{D_{\mathrm{dry}}}\times 100$$

Here, *D*_dry_ is the diameter of the dry sample, and *D*_swollen_ is the diameter of the swollen sample [21]. For each resin, three cylindrical discs were printed with a diameter of 9 mm and a thickness of 1 mm, before they were swollen in PBS buffer for 24 h. To monitor the potential release of unpolymerized components, 100 µL of the surrounding aqueous media were taken at different time points. Their absorbance was determined using a plate reader (TECAN infinite M200 PRO, Männedorf, Switzerland) in a range from λ = 350 nm to λ = 800 nm. A solution of pure PBS served as a blank sample.

### 2.8. Cell Culture

For all cell culture studies, primary HUVECs were used. For cultivation, cells were grown in tissue culture flasks (T-75), while exchanging the HUVECs culture medium supplemented with penicillin/streptomycin (1% *v/v*) every two to three days. The cells used for experiments were between passages three to six.

To test the effect of exposure of HUVECs to materials that were 3D-printed from our resin library, HUVECs were seeded inside a conventional PS 48-well culture plate, which was coated with fibronectin beforehand by incubating 200 µL fibronectin solution (50 mg mL^−1^ in PBS buffer) at room temperature for 30 min, followed by washing with PBS. Then, 3D-printed parts made from different RCs were sterilized by exposure to UV-light for 20 min before they were added to the PS wells. As a control, HUVECs were cultivated in the absence of any printed part inside PS 48-well culture plates. The cell morphology was assessed visually using an inverted phase-contrast microscope (Olympus IX73, Shinjuku, Japan) at different time points during cell culturing.

In a second experiment, to remove any potentially harmful remains of the resins diffusing out of the 3D-printed material into the surrounding medium, printed parts were kept in PBS buffer for two days, while exchanging the buffer on a daily basis. In the third set of experiments, the washing process was increased from two to four days.

To study the adhesion of HUVECs on our 3D-printed materials, small films of RC4, RC8, and RC9—which show no cytotoxicity—were prepared. These were subsequently coated with fibronectin via the same procedure as described for PS above to provide cell-anchoring points and promote the adhesion of HUVECs to a 3D-printed surface. HUVECs were then cultured for 24 days and again examined visually with a phase-contrast microscope. Further, immunofluorescence staining was performed. The specimens were fixed with paraformaldehyde solution (4% *v/v*) in PBS at room temperature for 20 min and washed. Then, the cells were exposed to a quenching solution (200 mM glycine, Triton X-100 at 0.3% *v/v* in PBS) for another 20 min. After quenching and additional washing with Triton X-100 at 0.3% (*v/v*) in PBS, a blocking solution (bovine serum albumin at 5% *v/v* with Triton X-100 in PBS at 0.3% *v/v*) was applied for 1 h to avoid unspecific binding. Finally, the specimens were stained with the blocking solution containing DARQ5™ (1:200) and phalloidin coupled with ATTO 550 dye (1:200). The staining solution was removed after 1 h of incubation, and the specimens were washed with the blocking solution twice to remove unbound dye. The resin films with adherent cells were taken from the multiwall plate and then placed between two glass slides to ensure that only cells adhering to the resins were visualized. The control sample in the multi-well plate and the resin samples were examined with a confocal spinning disk microscope (CSDM) (Andor Dragonfly, Andor Technology Belfast, Northern Ireland).

To investigate the efficiency of resin coating, we used fluorescently labeled fibronectin. The surfaces of the resins and PS were incubated with the blocking solution for 1 h. Then, a primary antibody (rabbit anti-fibronectin, 1:200 in blocking solution) and, subsequently, after washing, the secondary antibody (goat anti-rabbit, coupled to ATTO 647, 1:200 in blocking solution) were added. Again, both incubations were performed for 1 h. The samples were examined with the same setting for all samples (27% laser intensity, gain set to 622 V) using a confocal microscope (Leica TCS SP5, Leica, Wetzlar, Germany).

### 2.9. Cell Viability Assay

To quantify the viability of HUVECs cultured inside 3D-printed vessels made from RC4, RC8, and RC9, a viability assay was performed. These vessels had the same growth area as the PS-based control (48-well plate). They were washed with PBS or with Tween 20 prior to the cell culturing experiments, as described above. All surfaces (3D-printed and control) were coated with fibronectin. Approximately 10,000 cells were seeded per control well or 3D-printed vessel by adding 100 µL of a corresponding cell suspension. The cells were allowed to sediment and adhere for 2 to 3 h before more culture medium was added. The 3D-printed vessels were kept in a larger PS 24-well plate during the cultivation process and then transferred to a fresh multi-well plate before the viability assay was performed. This way, we ensured that only cells adherent to the printed surface were evaluated. These HUVECs were then cultured for 3 and 5 days, respectively. The viability was determined for triplicates of each experimental condition using PrestoBlue™ Cell Viability Reagent. First, 500 µL of reagent solution (10% *v/v*) in the cell culture medium was added into each well and incubated for 1 h at 37 °C, including a blank (growth medium with reagent). Then, the absorption spectra of 100 µL taken from the supernatant were measured at a wavelength of λ = 600 nm while applying the same gain for characterization both after 3 and 5 days.

## 3. Results and Discussion

Cell adhesion to (3D-printed) material surfaces requires tailoring biocompatibility, surface roughness, and wettability as key parameters. The number of commercial resins that fully address these is currently rather limited, however. On this account, we prepared a library of homemade resins based on PEGDA 250, PEGDA 575, TPGDA, POEA and PEGMEMA (cf. Table 1), and initially studied the contact angle toward aqueous media, their swelling in PBS buffer, the surface roughness of as-fabricated objects and their mechanical properties as well as the dose calibration required for optimal 3D-printing.

### 3.1. Material Characterization

The materials specifically chosen for this work have been investigated and used in biological applications outside the field of 3D printing previously. Low molecular weight acrylates such as PEGDA 250 and PEGDA 575 have been used for cultivating mammalian cells and in tissue engineering [22]. In another example, it has been shown that polymerized PEGDA has low binding toward proteins [23]. TPGDA has been used in non-cytotoxic acryl bone cement with antimicrobial properties [24]. However, these examples all lack a detailed study of biocompatibility and cytotoxicity to make use of them as 3D-printable cell culture platforms.

**Figure 1:**
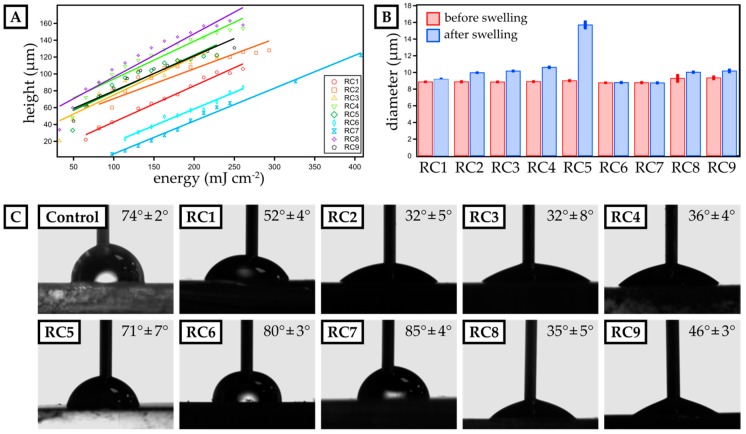
Characterization of dose calibration as well as swelling behavior and surface contact angle of micro-stereolithography (µSL)-printed, disk-shaped test objects. (**A**) The height of single 3D-printed layers is plotted against the exposure energy for resin compositions RC1 to RC9. A linear fit visualizes the data trend and is employed to calculate the exposure energy required for a distinct layer thickness. (**B**) The diameter of 3D-printed discs (three prints per resin) is measured before and after swelling in phosphate-buffered saline (PBS) buffer for 24 h for each resin composition. (**C**) The advancing contact angle of water is measured for 3D-printed layers made from resins RC1 to RC9. Polystyrene (PS) serves as a reference surface. The mean contact angle and the deviation for three individual experiments are included.

On this account, we prepared photopolymer formulations of these yet promising materials (cf. Table 1) and optimized them for µSL printing. First, the dose needed for a certain layer height was determined (Figure 1). For that, resin spots with a diameter of 3 mm were exposed to UV-light with a set intensity of 16.3 mW cm^−2^ for different exposure times (ranging from 1 to 20 s). The height of the spots was measured by confocal microscopy and plotted against the exposure time. Applying a linear fit, the dose needed for obtaining a certain layer thickness was determined. It was noteworthy that the addition of PEGMEMA to PEGDA-based resins increased the minimum amount of energy to form a polymerized spot. For RC1 made from PEGDA 250, a minimum energy of 62 mJ cm^−2^ was sufficient to polymerize a spot, but for RC2, 82 mJ cm^−2^ was required. The same trend could be observed from RC3 (pure PEGDA 575) to RC4 (PEGDA 575 with PEGMEMA). Again, more energy was required to build up a mechanically sTable 3D-printed spot. This observation was consistent with established literature on radical polymerization of acrylates, where it has been discussed that methacrylates are less reactive than acrylates [19]. Furthermore, PEGMEMA is a monofunctional acrylate, which leads to a lower reactivity compared to pure bifunctional PEGDA. Since RC8 and RC9 were made out of the same oligomers as RC4, but with different percentages per weight, their spot height was similar as that found for RC4. The height measured for RC6 and RC7, which contained TPGDA (RC6) and TPGDA combined with POEA (RC7), was half the height of the PEGDA and PEGMEMA-based resins.

Next, we studied the swelling in aqueous media of 3D-printed discs with a diameter of 9 mm and a height of 1 mm (Figure 1B) fabricated from our resin library. Assessing the swelling behavior is crucial to ensure structural stability in cell culture media during long-term growth. Generally, PEGMEMA-based resins showed significant swelling in water, e.g., RC5 exhibiting a swelling ratio of 74.1%. By preparing mixtures of PEGDA and PEGMEMA (RC1 to RC4), we drastically reduced the swelling ratio varying from 3.5% (RC1) up to 19.3% (RC4). Here, replacing PEGDA 250 by the slightly larger PEGDA 575, led to an increase in polymer network spacing, thus increasing the ability to absorb water. Finally, TPGDA-based resins showed the lowest swelling ratio with less than 0.5% over a period of 24 h in PBS.

To ensure optimal contact between the cell media and a 3D-printed cell culture substrate, we studied the contact angle of water and compare it to PS as our reference material, which is commonly used as a cell culture substrate. While the contact angle for PS was 74° ± 2°, only RC5, RC6 and RC7 showed a similar contact angle, while resins based on PEGDA (RC1 to RC4) were more hydrophilic with contact angles ranging from 32° ± 5° up to 52° ± 4°. By replacing PEGDA by PEGMEMA, we increased the contact angle further up to 71° ± 7° (RC5). Eventually, TPGDA-based and TPGDA/POEA-based resins were more hydrophobic and, accordingly, we measured contact angles above 80° (RC6 & RC7).

Finally, the surface roughness was determined (Figure S1) following the norm ISO 4287. Lampin et al. previously showed that surface roughness of more than 180 nm can be advantageous for the adhesion of vascular cells [20]. In addition, materials with low surface energy and surface roughness of 500 nm and higher may enhance cell adhesion [25]. Thus, we assessed the surface roughness based on the arithmetic mean deviation (*R*_a_) of 10 profiles for each resin composition. We particularly focused on the last printed layer, as this would be exposed to cell suspensions in all later experiments. The obtained values varied between 250 ± 20 nm (RC2) and 740 ± 80 nm (RC7). Again, compared to PS (*R*_a_ = 20 ± 0 nm), the values were one magnitude larger, but comparable to Lampin et al.

### 3.2. Cytotoxic Effect on Human Umbilical Vein Endothelial Cells (HUVECs)

**Figure 2:**
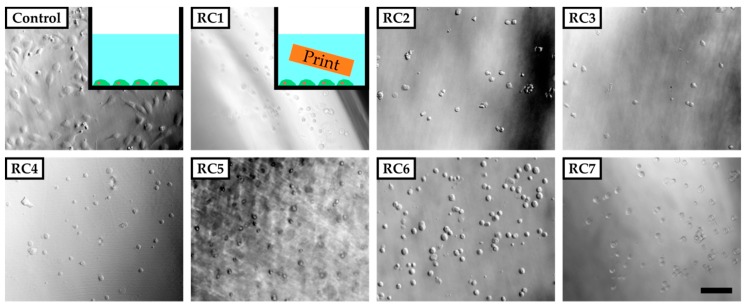
Phase-contrast images of human umbilical vein endothelial cells (HUVECs) five hours after seeding in poly(styrene) (PS) culture vessels coated with fibronectin. The cells adhere and spread on the surface of the vessel in the absence of any 3D-printed parts (inset top left corner). In the presence of 3D-printed test objects made from resins RC1 to RC7 added to the vessel, cells do not adhere to the surface of the PS vessel but remain in solution with a spherical shape. The scale bar denotes 200 µm. In Section 3.1, it was shown that the 3D-printed parts tend to swell and therefore could potentially release substances into the culture medium that are not covalently bound to the polymer network.

To evaluate the cytotoxicity of our resins, HUVECs were grown in PS 48-wells in the presence of a 3D-printed block of each resin in cell culture media (Figure 2, inset). For that, the PS-based cell culture plate was coated with fibronectin to ensure sufficient binding of HUVECs, which were then exposed to 3D-printed parts made from RC1 to RC7 inside these wells. RC8 and RC9 were not tested in these preliminary experiments since they contained the same components as RC4. Images were recorded 5 h after cell seeding. Only in the absence of 3D-printed parts did cells adhere to the surface of the PS 48-well and start to spread. For all experiments involving 3D-printed parts inside the cell culture media, the cells did not adhere to the surface and remained spherical in solution. The results clearly showed that the cells were not viable in the presence of 3D-printed objects made from any of the resins under investigation at this point. We assume that the 3D-printed materials release cytotoxic components from the initial photopolymer formulation into the cell culture medium and prevent the cells adhering to the PS surface or spreading.

On this account, it was vital to identify, which components are potentially released from the 3D-printed parts during application and contribute to the observed cytotoxicity. While TPO—acting as a photo-absorber—was present in all our resins, previous studies on the usage of TPO or its derivative (ethyl 2,4,6-trimethylbenzoylphenylphosphinate) to establish artificial extracellular environments showed that TPO can be safely used and should not influence cell viability, even when used at concentrations as high as 2% *w/w* compared to concentrations ranging from 0.1% to 0.25% in our resins [26,27]. Uncured resin, as another source for the observed cytotoxicity, should have been consumed in the post-curing process, where 3D-printed objects were exposed to UV-light to achieve complete conversion, and in the sterilization process, which also involved UV-light.

Frei et al., 2002. and Barfknecht et al., 1985 showed that Sudan 1, which is used as the UV-absorber in the resin formulations, is suspected to be genotoxic through the formation of radical species [28,29]. A higher swelling ratio of 3D-printed polymer materials should also correlate with higher release rates of Sudan 1.

To avoid the release of Sudan 1 and minimize its harmful effect on cells, which is also considered to be dose-dependent in vitro [30], 3D-printed parts were washed with PBS buffer for four days—with a daily exchange of buffer—prior to exposing them to HUVECs. To detect the presence of Sudan 1 in the supernatant emerging from the 3D-printed materials, absorption spectra of the PBS buffer were recorded at different time intervals and compared to pure Sudan 1 (Figure S2). The characteristic maximum absorbance wavelength of Sudan 1 was λ = 480 nm. For all samples (RC1 to RC7), the absorbance increased over time within an interval of 1 h to 5 days. In particular, RC3, RC4 and RC5 released Sudan 1 indicated by a remarkable increase in absorption in the range from λ = 450 nm to λ = 500 nm [31].

**Figure 3:**
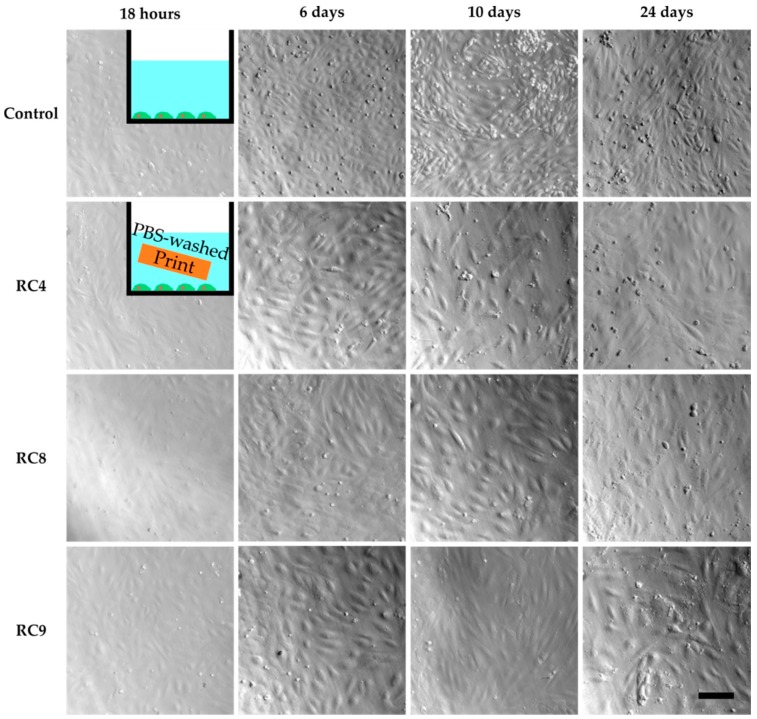
HUVECs cultivated in PS 48-wells for 24 days, with images taken after 18 h, 6 days, 10 days and 24 days to study cell viability. The first image row shows the control experiment without the presence of any 3D-printed parts in the cell culture media. Row two to four show images of HUVECs exposed to 3D-printed parts made from RC4, RC8, and RC9, respectively. The HUVECs adhere to the PS surface, spread, and are viable as seen from 18 h to 24 days. In addition, the cell density is similar to the control. The scale bar denotes 200 µm for all samples.

However, cell culture experiments with HUVECs in the presence of washed 3D-printed objects showed similar results as the previous cell culture studies in the presence of untreated samples (Figure S3). Only for RC4, cells adhered to the PS surface while being exposed to a 3D-printed block made from RC4 and showed the same morphology as the control two days into culturing. In addition, the cell density was comparable between the control and RC4. To verify these findings, a long-term experiment was performed. HUVECs are cultivated for 24 days in the presence of RC4 and two variations of RC4 with a higher amount of PEGDA 575, namely RC8 and RC9 (Figure 3). The cells adhered to the PS surface and spread similarly to the control. Again, a comparable cell density was observed. It was concluded that 4 days of washing is sufficient to avoid a cytotoxic effect, depending on the resin formulation.

Next, we investigated if HUVECs were able to grow on the surface of 3D-printed samples made from our resin library. For that, layers of RC4, RC8, and RC9 were exposed to UV-light and coated with fibronectin (cf. Section 2.9), as it is known that the RGD tripeptide (arginine, glycine, and aspartate) within fibronectin is a crucial sequence for promoting cell adhesion, e.g., of endothelial cells [32]. HUVECs were cultivated for 3 days on the PS control, RC4, RC8, and RC9, respectively. Corresponding phase-contrast microscopy images are shown in Figure S4. On all samples, cells were adherent and spread, which indicated there was no cytotoxic effect of the 3D-printed materials. As before, the morphology of the cells was similar to cells grown on the PS control. However, we found it challenging to visualize the cells on the 3D-printed materials due to their rough surface. Only for RC9, the image quality was comparable to the PS control. Besides, cells grown on the resin-based materials did not spread homogenously. Instead, patches of HUVECs were observed in some areas whereas there are no cells in other areas. Thus, the overall cell density was lower. We attributed this observation to two reasons. First, the direct contact of the 3D-printed material with the cells could have harmed the cells lowering the numbers of cells growing on the surface. It is known that PEGDA-based hydrogels while being non-cytotoxic, resist cell adhesion [33,34]. The same effect could occur on 3D-printed PEGDA-based materials just like on those made from RC4, RC8, and RC9. Second, Burdick and Anseth showed a bioactive linker molecule is required for sufficient cell adhesion on PEG-based substrates [35]. Although we applied fibronectin to the surface of all 3D-printed samples, it is hypothesized at this point based on phase-contrast microscopy characterization that the coating was not sufficiently stable during long-term culturing or homogenous to provide an adequate number of binding sites for HUVECs.

**Figure 4:**
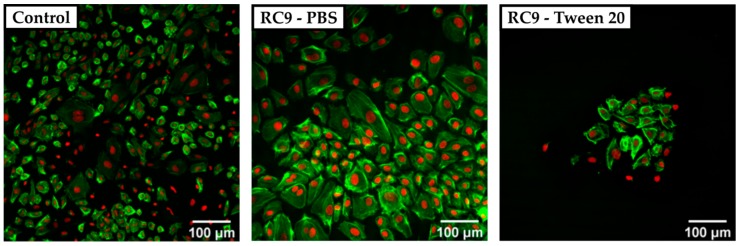
Confocal spinning disk microscope (CSDM) images of HUVECs grown on a PS control substrate and on RC9-based substrate washed with PBS and Tween 20. The nuclei are stained with DARQ5 (red), and the actin is marked with phalloidin coupled with ATTO 550 dye (green). The control sample shows a nearly confluent layer of cells with a heterogeneous morphology of widely spread and small cells (left). Cells cultivated on PBS-washed, RC9-based samples show a similar morphology but at lower density (middle). Washing with Tween 20 leads to a remarkably lower number of cells with locally similar cell density as in the control (right).

To circumvent the poor visualization in phase-contrast microscopy regarding surface roughness, we utilized CSDM. As the broad absorbance spectrum of Sudan 1 limited the choice of fluorescence dyes for cell structure visualization, we utilized DARQ5 and ATTO 550. Fluorescence images showed large Sudan 1 crystals that covered the surface of RC4, RC8, and RC9 and exacerbate a clear visualization of HUVECs. Therefore, resin-based substrates were washed beforehand with PBS and also with PBS containing the detergent Tween 20 at 1% (*v/v*). HUVECs were cultivated for three days and visualized by CSDM, as shown for RC9 (Figure 4). Here, the nuclei appear in red (DARQ5), and the actin filaments in green (phalloidin coupled with ATTO 550 dye).

The cell density on any of the tested 3D-printed substrates was lower compared to the PS control sample. Cells cultivated on the control surface showed a heterogeneous morphology with widely spread cells. In samples washed with Tween 20, no crystals of Sudan 1 were found on the surface, but the number of adherent cells was remarkably lower compared to the control and 3D-printed samples treated with PBS only prior to cell culture. It was considered that the detergent was not removed completely and therefore, the remains of Tween 20 may have harmed the cells by solubilizing lipids from their membrane [36]. Adding multiple washing steps could potentially avoid the insufficient removal of detergent and increase the number of adherent cells.

### 3.3. Cell Viability Assay

**Figure 5:**
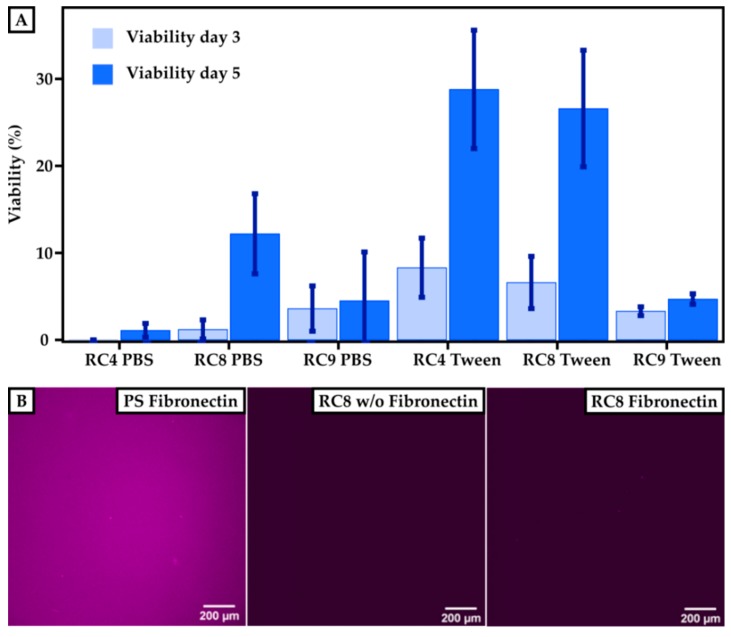
Cell viability on 3D-printed surfaces compared to PS-based control substrates. (**A**) Results of the cultivation of HUVECs on a PS control are set to 100%. The viability is measured after 3 and 5 days for printed samples of RC4, RC8, and RC9 washed either with PBS or with Tween 20. The viability increases for each sample after 5 days. Washing the samples with Tween 20 in PBS instead of pure PBS generally leads to higher cell viability. (**B**) To investigate the efficiency of the fibronectin coating, images of the PS control and RC8 without and with coating are presented. The presence of immunofluorescently labeled fibronectin is indicated by a purple color. Homogeneous coating of fibronectin can be seen for the PS control, but for RC8 no fibronectin is detected, which implies that the coating has not been successful.

To determine the cell viability on 3D-printed materials, HUVECs were cultured for 3 and 5 days, respectively, and compared to PS control substrates. The viability of the cells was measured using a resazurin-based viability test, which monitored the cell’s metabolic activity. Here, the fluorescence absorbance of resorufin (note: resazurin is reduced to resorufin by metabolic activity) in the supernatant of the cell culture correlated with the viability of the cells. The fluorescence signal observed for the control samples after 3 days was set to 100% viability. The cell viability for HUVECs grown on 3D-printed materials made from RC4, RC8, and RC9 was measured and set in relation to the control, as shown in Figure 5A. All samples showed that the viability of HUVECs after 3 days was higher when treating the 3D-printed substrates with Tween 20 prior to usage. The fluorescence signal for 3D-printed parts made out of RC4 and washed with PBS was under the detection limit and set to 0% after 3 days since no metabolic activity was measured. Here, the viability after 5 days of culturing increased for substrates made from RC4 and RC8. For PBS-washed samples made from RC4, the increase from 3 to 5 days was merely 1.1% ± 0.8%. However, the increase in cell viability on the same samples washed with Tween 20 in PBS was much more pronounced with values ranging from 8.3% ± 3.4% (3 days) to 28.8% ± 6.8% (5 days). Similar results were obtained for RC8-based 3D-printed parts. While the viability was relatively low for PBS-treated samples 1.2% ± 1.1% up to 12.2% ± 4.6%), it rapidly increased from 6.6% ± 3.0% (3 days) to 26.6% ± 6.7% (5 days) when washing the 3D-printed cell culturing substrates with detergent beforehand, indicating that the cells were viable and the 3D-printed substrates non-cytotoxic. Only for RC9-based samples, a treatment with Tween 20 (3.6% ± 2.6% after 3 days) did not show any significant increase in viability compared to washing it with PBS (3.3% ± 0.5% after 3 days).

While the resins showed cell compatibility, we attributed the rather low cell viability to an insufficient coating of the 3D-printed parts with fibronectin and thus low binding of HUVECs to the surface. The smaller number of cells adhering to the 3D-printed material’s surface may have resulted from a lack of binding sites necessary to promote cell adhesion. Therefore, we investigated the efficiency of the fibronectin coating. The coating procedure worked well for PS surfaces, as shown in Figure 5B. The purple color indicates the presence of fibronectin, which is homogeneously distributed over the whole substrate. However, the coating of 3D-printed surfaces with fibronectin—shown, for example, for RC8—was unsuccessful which we attributed to the low protein binding of PEGDA [23]. Thus, the binding of HUVECs on the surface was not promoted, and a lower amount of HUVECs adhered to the surface in the first line. However, we are confident that the tested 3D-printed substrates made from resins RC4, RC8, and RC9 were non-cytotoxic, referring to the significant increase in viability from 3 to 5 days of culture. Here, the cells grew and proliferated even in the absence of fibronectin coating.

## 4. Conclusions

Resin formulations for µSL-based printing and subsequent cultivation of HUVECs have been developed. By screening material properties such as dose calibration, contact angle toward aqueous solutions, swelling degree in aqueous buffer, and surface roughness, we determined key parameters required for applying 3D-printed materials in cell culture experiments. The tested materials were PEGDA, PEGMEMA, TPGDA, and POEA and were used in (cell-)biological applications previously, but without testing the cytotoxic effect of resins for 3D printing made from these materials. Using three examples from our resin library, we showed that a combination of PEGDA with PEGMEMA (and Sudan 1 as the cross-linker, and TPO as the photo-initiator) can be used to 3D-print surfaces for the cultivation of cells. By implementing post-processing steps that involve washing of printed materials with detergent in PBS before cultivation, unpolymerized substances can be washed out that otherwise harm the cells. Here, our experiments indicated that insufficient removal of detergent directly reduces the attachment of HUVECs on the surface. We proved that the release of Sudan 1 from the 3D-printed materials correlates with their swelling behavior and is also a key component in the resin formulation responsible for the cytotoxicity of 3D-printed materials that are not post-processed. Finally, the viability of HUVECs grown on 3D-printed materials was investigated. Even though the viability after 3 days of cultivation was limited, the cells significantly proliferated within two additional days of culture on PEGDA/PEGMEMA-based surfaces, which proved their non-cytotoxicity. Compared to the PS standard, we achieved initial viability of 28.8% ± 6.8%, which we think will be greatly improved by further refining the incorporation of cell anchoring points, e.g., by incorporation of RGD tripeptides on the resin level rather than by post-processing 3D-printed surfaces with fibronectin.

## Figures and Tables

**Table 1:** The resin composition of nine different resins containing monomers/oligomers, crosslinker, ultraviolet (UV)-absorber, and initiator.

Resin Composition (RC)	Monomer/Oligomer (% *w/w*)		Crosslinker (% *w/w*)	UV-Absorber (% *w/w*)	Initiator (% *w/w*)
RC1	PEGDA 250	99.65	-	0.25	0.10
RC2	PEGDA 250 PEGMEMA 500	44.83	10.00	0.25	0.10
Wo RC3	PEGDA 575	99.65	-	0.25	0.10
RC4	PEGDA 575 PEGMEMA 500	44.83	10.00	0.25	0.10
RC5	PEGMEMA	89.50	10.00	0.25	0.25
RC6	TPGDA	99.65	-	0.25	0.10
RC7	POEA TPGDA	69.65 30.00	-	0.25	0.10
RC8	PEGDA 575 PEGMEMA 500	69.65 20.00	10.00	0.25	0.10
RC9	PEGDA 575 PEGMEMA 500	79.65 10.00	10.00	0.25	0.10

## Supplementary Materials

The following are available online at https://www.mdpi.com/2072-666X/11/3/246/s1: Figure S1: Surface roughness of printed materials, Figure S2: Release of Sudan 1 from printed materials, Figure S3: Cell culture studies on 3D-printed materials washed for four days with PBS buffer, Figure S4: HUVECs cultivated on 3D-printed materials imaged with phase-contrast microscopy.
