# Supplementary material for: A Non-Cytotoxic Resin for Micro-Stereolithography for Cell Cultures of HUVECs
Source: Micromachines (Basel). 2020 Feb 26;11(3):246. doi: 10.3390/mi11030246 (PMC7143370; doi:10.3390/mi11030246)
Supplement: Supplementary file 1 [file micromachines-11-00246-s001.pdf]

micromachines

A Non-Cytotoxic Resin for Micro-Stereolithography for Cell Cultures of HUVECs

Max J. Männel, Carolin Fischer and Julian Thiele*

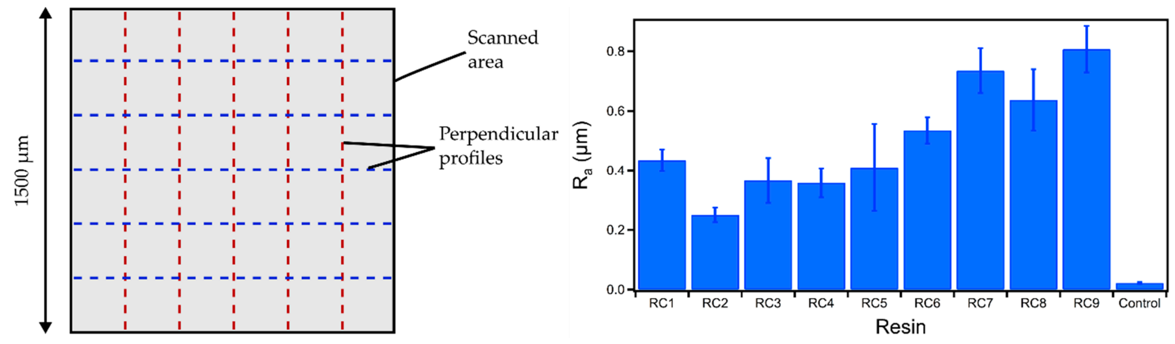

Figure S1. To measure the surface roughness of 3D-printed test objects, 10 cross-sections were measured over 1500 µm whereas five cross-sections were perpendicular to the others (**left**). The roughness was determined for each resin composition (**right**). Compared to the control polystyrene (PS), the surface roughness R_a of the resins was approximately one magnitude larger.

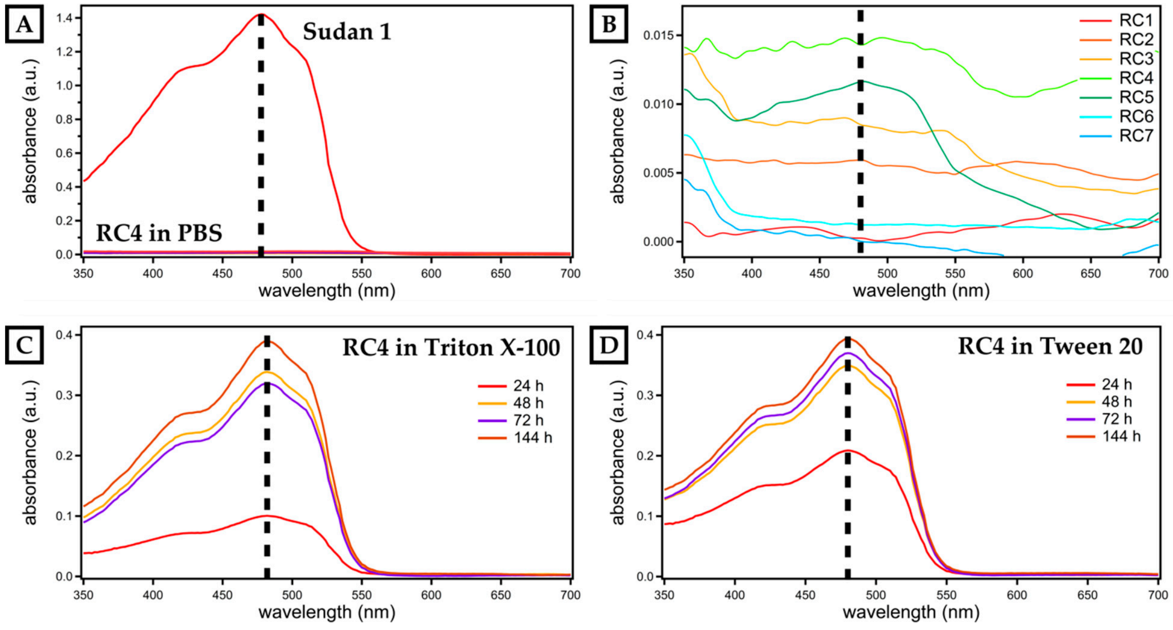

Figure S2. To measure the release of Sudan 1 from µSL-printed test objects, ultraviolet-visible (UV-Vis) spectra of PBS buffer samples were recorded that were used to wash µSL-printed polymer materials made from RC1 to RC7. The absorbance maximum of Sudan 1 at $\lambda = 480$ nm is indicated by a dashed line in all spectra. (**A**) Absorption spectrum of Sudan 1 in PBS (red) compared to PBS buffer after treating 3D-printed test objects made from PEGDA 250 and RC4. (**B**) UV-Vis spectra of PBS buffer after treating 3D-printed parts made from RC1 to RC7. The highest absorption was found for RC3, RC4, and RC5. (**C,D**) The absorbance was measured for RC4 washed with Triton X-100 in PBS, and Tween 20 in PBS, respectively, for time intervals from 24 hours to 144 hours.

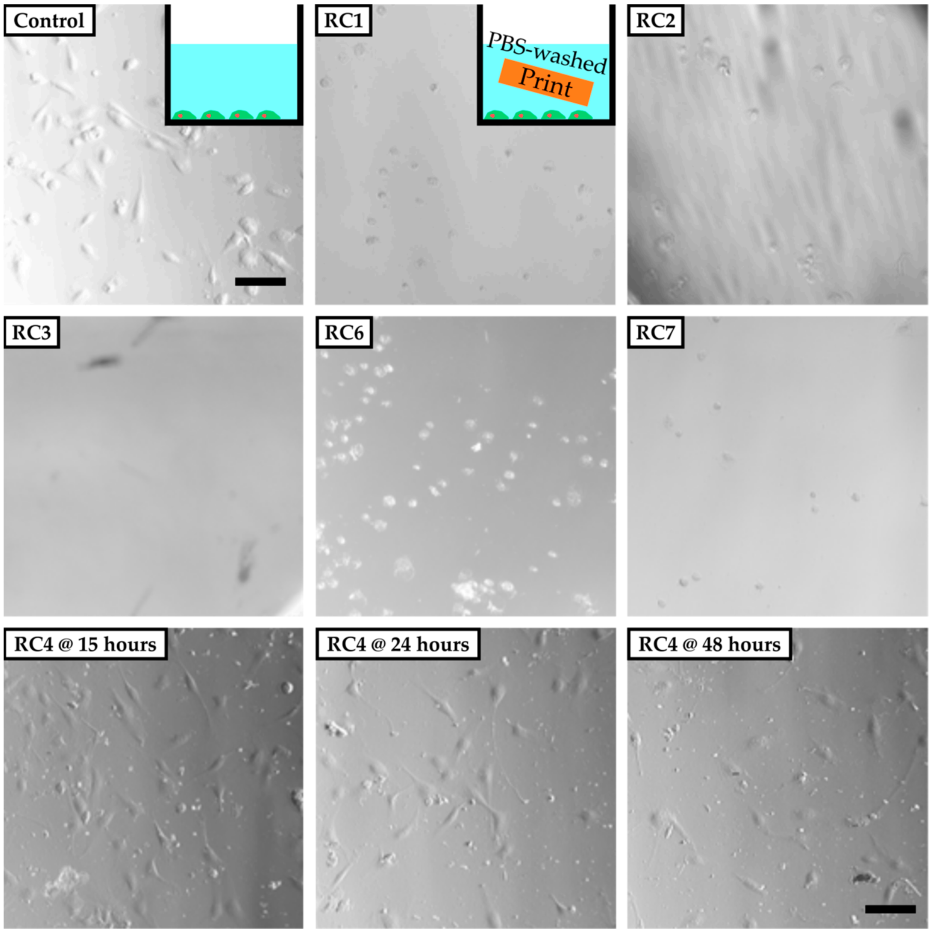

Figure S3. Cell culture experiments on PS substrates in the presence of 3D-printed parts treated only with PBS buffer before transfer into culture medium. HUVECs were adherent to the surface and spread for the control and for RC4 (last row). For all other samples, the cells did not adhere to the surface and remained spherical. Based on these findings, two more resin formulations (RC8 and RC9) were prepared similar to RC4, which were non-cytotoxic (Figure 3). The scale bar denotes 200 μm for all images.

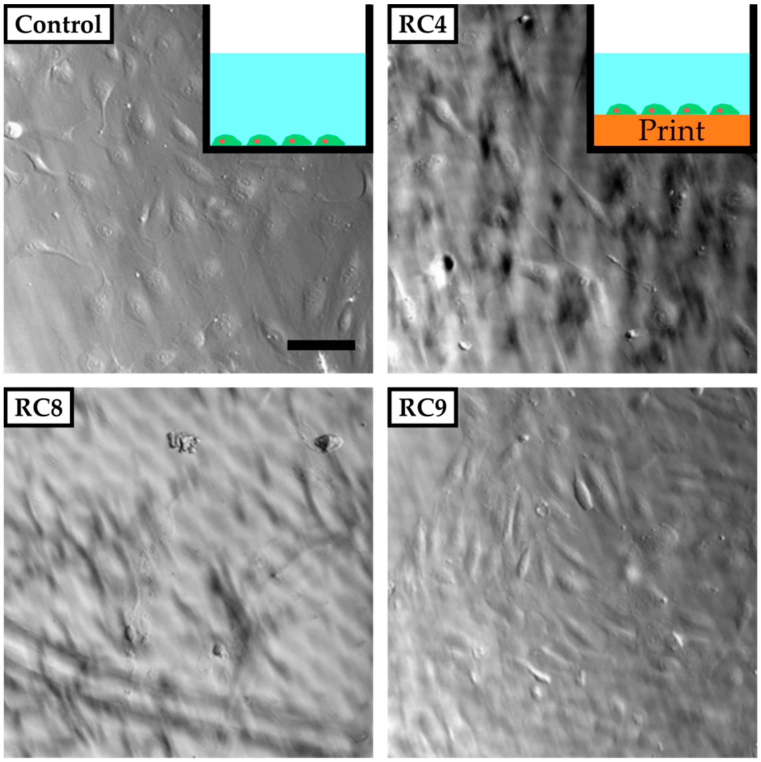

Figure S4. HUVECs cultivated on the surface of the PS-based control samples, and compared to 3D-printed cell culturing surfaces made from RC4, RC8, and RC9. The cells were adherent to all four tested surfaces and spread, also exhibiting similar cell morphology among all samples. The scale bar denotes 200 μm for all panels.
